# HT-29 and Caco-2 Reporter Cell Lines for Functional Studies of Nuclear Factor Kappa B Activation

**DOI:** 10.1155/2015/860534

**Published:** 2015-03-10

**Authors:** Giuliana Mastropietro, Inés Tiscornia, Karen Perelmuter, Soledad Astrada, Mariela Bollati-Fogolín

**Affiliations:** Cell Biology Unit, Institut Pasteur de Montevideo, Mataojo 2020, 11400 Montevideo, Uruguay

## Abstract

The NF-*κ*B is a transcription factor which plays a key role in regulating biological processes. In response to signals, NF-*κ*B activation occurs via phosphorylation of its inhibitor, which dissociates from the NF-*κ*B dimer allowing the translocation to the nucleus, inducing gene expression. NF-*κ*B activation has direct screening applications for drug discovery for several therapeutic indications. Thus, pathway-specific reporter cell systems appear as useful tools to screen and unravel the mode of action of probiotics and natural and synthetic compounds. Here, we describe the generation, characterization, and validation of human epithelial reporter cell lines for functional studies of NF-*κ*B activation by different pro- and anti-inflammatory agents. Caco-2 and HT-29 cells were transfected with a pNF-*κ*B-hrGFP plasmid which contains the GFP gene under the control of NF-*κ*B binding elements. Three proinflammatory cytokines (TNF-*α*, IL-1*β*, and LPS) were able to activate the reporter systems in a dose-response manner, which corresponds to the activation of the NF-*κ*B signaling pathway. Finally, the reporter cell lines were validated using lactic acid bacteria and a natural compound. We have established robust Caco-2-NF-*κ*B-hrGFP and HT-29-NF-*κ*B-hrGFP reporter cell lines which represent a valuable tool for primary screening and identification of bacterial strains and compounds with a potential therapeutic interest.

## 1. Introduction

Gut epithelium is critically involved in the maintenance of intestinal immune homeostasis acting as a physical barrier separating luminal bacteria and immune cells and also expressing antimicrobial peptides. Transcription factor NF-*κ*B, a master regulator of proinflammatory responses, works in gut epithelial cells in order to control epithelial integrity and the interaction between the mucosal immune system and gut microbiota [1]. Gut epithelial cells have the ability to act as frontline sensors for microbial encounters and to integrate commensal bacteria-derived signals into antimicrobial and immunoregulatory responses. They express pattern-recognition receptors that enable them to act as dynamic sensors of the microbial environment and as active participants in directing mucosal immune cell responses [2].

NF-*κ*B signaling is triggered in a series of steps through either the classical canonical pathway, the alternative noncanonical pathway, or the atypical I*κ*K independent pathway [3]. NF-*κ*B is typically present and resides in the cytoplasm of most cells as a complex with members of the I*κ*B inhibitor protein family. Both the size of this complex and I*κ*B's masking of the nuclear localization sequence of NF-*κ*B prevent NF-*κ*B from entering the nucleus through the nuclear membrane. Once I*κ*B is phosphorylated and degraded, the nuclear localization sequences become accessible and NF-*κ*B is free to translocate into the nucleus [4]. Translocation of NF-*κ*B is a critical step in the coupling of extracellular stimuli to the transcriptional activation of specific target genes. Over 200 physiological stimuli are known to activate NF-*κ*B, for instance, proinflammatory cytokines (IL-1*α*, IL-1*β*, and TNF-*α*); bacterial toxins (LPS, exotoxin B); viral products (HIV-1, HTLV-1, HBV, EBV, and Herpes Simplex); and cell death stimuli (O_2_-free radicals, UV light, and *γ*-radiation) [3, 5]. The major cellular targets of NF-*κ*B are chemokines, immune receptors, adhesion molecules, stress response genes, regulators of apoptosis, transcription and growth factors, enzymes, and cell cycle regulators [3].

The NF-*κ*B activation has direct screening applications for immunomodulatory compounds and potential probiotic strains. It is essential to have accurate and reproducible techniques that measure the activation or inhibition of NF-*κ*B. Conventional methods are based on determining the localization of NF-*κ*B as a parameter of activation, which can be determined by western blotting, gel shift assay, or microscopy of immunolabeled cells. In this sense, Trask reported a high content assay, an automated fluorescent microscopy computer-assisted image analysis technology, for running a compound screening campaign that quantifies the redistribution of NF-*κ*B from the cytoplasm to the nucleus upon activation [6]. Antibodies against subunit p65 of NF-*κ*B were employed on HeLa cells following fixation. Cell imaging provides multiprobe detection and is advantageous in quantifying spatial measurements, and compared to western blotting, it provides information of the heterogeneity of the sample. However, as a disadvantage, cells require tedious processing, including fixation. Moreover, it lacks sensitivity in detecting rare events within a sample. ImageStream Cytometry has been proposed as a technique that overcomes these limitations but requires a specific and expensive instrument [7].

Due to all the above-mentioned impediments, the generation of reporter cell lines to monitor NF-*κ*B activation became an attractive tool. Lakhdari and colleagues generated stable cell lines for NF-*κ*B activation using a secreted alkaline phosphatase reporter gene and performed a high throughput screening of a metagenomic library of Chron's patients microbiota [8]. Badr and colleagues developed a reporter system based on naturally secreted* Gaussia* luciferase, in different models including tumors, angiogenesis, and inflammation [9]. None of these studies used a reporter protein such as Green Fluorescent Protein (GFP), which eliminates the need for an external substrate and does not require laborious sample processing. Here, we describe cellular tools for functional studies of NF-*κ*B activation in intestinal epithelial cells (IECs). Two reporter cell lines based on IECs, Caco-2, and HT-29 were generated, which express GFP as a reporter of NF-*κ*B activation. The response of these novel reporter cell lines was extensively characterized by flow cytometry and validated using lactic acid bacteria (LAB) with immunomodulatory properties [10, 11] and a natural peptide with anti-inflammatory properties [12].

## 2. Materials and Methods

### 2.1. Reagents

Unless otherwise indicated, all chemicals used were of the highest available grade and purchased from Sigma Aldrich. Culture media, fetal bovine serum (FBS), and consumables for cell culture were obtained from Life Technologies, GE Healthcare, and Greiner. IL-1*β*, LPS, and TNF-*α* were purchased from R&D (USA) and Sigma (USA).

### 2.2. Cell Lines and Culture Medium

Caco-2 (ATCC HTB-37) and HT-29 (ATCC HTB-38) cells were cultured in RPMI1640 (Life Technologies, USA) or DMEM (Life Technologies, USA) and supplemented with 10% (v/v) FBS (Life Technologies, USA). Cells were routinely propagated in 25 or 75 cm^2^ tissue culture flasks at 37°C, 5% CO_2_ in a humidified incubator until reaching approximately 70% confluence. Subsequently, cells were trypsinized, concentration was adjusted, and cells were used for different experimental settings. In all described assays cells were cultured for less than twenty passages.

### 2.3. Generation of Stable Reporter Cell Lines

For reporter cell line generation 4 × 10^5^ cells were seeded in a 25 cm^2^ T-flask and transfected using 10 *μ*g of pNF-*κ*B-hrGFP plasmid (Agilent Technologies, USA) and Lipofectamine LTX (Life Technologies, USA) according to manufacturer instructions. Cell colonies were selected and expanded in medium containing hygromycin B (Sigma, USA) (50 *μ*g/mL) during forty five days. Resistant cells were stimulated with TNF-*α* (50 ng/mL) and those expressing GFP were sorted using a MoFlo XDP cell sorter (Beckman Coulter, USA) in “single cell” mode with a 0.5 drop sort envelope criteria. GFP excitation was achieved using a 488 nm Argon laser and fluorescence emission was detected employing a 530/40 band-pass filter. Sort decision was based on FSC versus SSC dot plots, excluding doublets and including GFP positive cells on FSC versus GFP fluorescence dot plots. Cells were placed into individual wells in a 96-well plate containing 100 *μ*L culture medium supplemented with 20% (v/v) FBS and penicillin/streptomycin. When clones reached confluence, they were evaluated with different stimuli which produced NF-*κ*B activation and GFP expression. This was assessed by flow cytometry (see section* Reporter Gene Assay Conditions*). Clones that showed the best response were amplified and cryopreserved.

### 2.4. Reporter Gene Assay Conditions

Caco-2-NF-*κ*B-hrGFP clones (C3 and D5) were seeded in 48-well plates (1 × 10^5^ cells/well) in DMEM supplemented with 10% (v/v) FBS. HT-29-NF-*κ*B-hrGFP clones (E5 and F6) were seeded in 96-well plates (5.0 × 10^4^ cells/well) in RPMI1640 media containing 10% (v/v) FBS. After 24 h, medium was renewed and different stimuli (TNF-*α*, IL-1*β*, or LPS) were added. Caco-2-NF-*κ*B-hrGFP and HT-29-NF-*κ*B-hrGFP cells were incubated for 48 or 18–24 h, respectively, at 37°C in a 5% CO_2_ humidified atmosphere. Finally, cells were trypsinized and resuspended for flow cytometry analysis. Cells were analyzed using a CyAn ADP (Beckman Coulter, USA) flow cytometer equipped with 488 nm and 635 nm lasers. Summit v4.3 software was used for data acquisition and FlowJo vX.0.7 for analysis. GFP and propidium iodide fluorescence emissions were detected using band-pass filters 530/40 and 613/20, respectively. For each sample, 10,000 counts gated on an FSC versus SSC dot plot, excluding doublets were recorded. Only single living cells (cells that excluded propidium iodide) were considered for results comparison.

### 2.5. Characterization of Reporter Cell Lines

#### 2.5.1. Time Course Kinetics

Caco-2-NF-*κ*B-hrGFP and HT-29-NF-*κ*B-hrGFP clones were seeded in 48-well or 96-well plates, respectively. After 24 h, medium was renewed, TNF-*α* (50 ng/mL) was added, and cells were incubated at 37°C and 5% CO_2_ for 0, 18, 24, 48, 72, 96, and 144 h for Caco-2 clones and for 0, 18, 24, 48, 72, and 96 h for HT-29 clones. Then, cells were trypsinized and GFP expression was analyzed by flow cytometry.

#### 2.5.2. Activation of NF-*κ*B with Different Stimuli

Caco-2-NF-*κ*B-hrGFP and HT-29-NF-*κ*B-hrGFP clones were seeded in 48-well or 96-well plates, respectively. After 24 h, medium was renewed, different stimuli (TNF-*α*: 0.004–100 ng/mL, IL-1*β*: 0.0016–25 ng/mL, or LPS: 0.0025–64 ng/mL) were added, and cells were incubated at 37°C in a 5% CO_2_ humidified atmosphere. Each concentration was assayed in triplicate. After 48 h (for Caco-2-NF-*κ*B-hrGFP) or 18–24 h (for HT-29-NF-*κ*B-hrGFP), cells were analyzed by flow cytometry. Dose-response curves were plotted expressing data as the mean of triplicates with standard deviation (SD) error bars. Curves were fitted using nonlinear regression 3-parameter fit using GraphPad Prism Version 5.00 (Trial) (GraphPad Software, Inc., USA). Detection limit was calculated considering the average signal value corresponding to the negative control plus 3 times the SD. Taking the dose-response curves into account, the assay linearity range was determined from the linear portion of each standard curve.

#### 2.5.3. Stability of the Reporter Cell Line

The stability of the reporter cell lines was verified at different passage numbers and with various stimuli for each cell clone. Thus, cell clones considered as time passage 0 were compared with cells which had undergone a weekly splitting for 1 or 2 months (passage 4 and 8, resp.). Cells from different passage numbers (0, 4, and 8) were seeded in 96- or 48-well plates and cultured overnight (ON). Caco-2-NF-*κ*B-hrGFP C3 clone and HT-29-NF-*κ*B-hrGFP E5 clone were stimulated with either TNF-*α* (0.004–1,000 ng/mL) or IL-1*β* (0.0016–25 ng/mL). HT-29-NF-*κ*B-hrGFP F6 clone was stimulated with TNF-*α* (0.004–100 ng/mL) and LPS (0.0025–64 ng/mL). Finally, cells were analyzed by flow cytometry at 48 and 18–24 h for Caco-2-NF-*κ*B-hrGFP and HT-29-NF-*κ*B-hrGFP, respectively. Data was normalized and EC50 was determined using nonlinear regression 3-parameter fit.

#### 2.5.4. Polarization of Caco-2-NF-*κ*B-hrGFP Cells

Cells were grown in 48-well plastic culture plates or in the upper chamber of a transwell filter (3 *μ*m diameter of pores; Costar, USA) and supplied with fresh culture media every 48 h. After 21 days postconfluence, medium was renewed and either TNF-*α* (0.05–100 ng/mL) or IL-1*β* (0.0016–10 ng/mL) were added, as described previously. For cells grown in transwell filter, only TNF-*α* was assayed and it was added in the basolateral chamber. Activation of NF-*κ*B was evaluated through flow cytometry.

To verify cell polarization, localization of nuclei and distribution of actin were determined. Cells were seeded in a 12-well plastic culture plate containing glass cover slips in DMEM supplemented with 10% (v/v) FBS. At day 21, culture media was removed and immediately after cells were fixed by adding 500 *μ*L of 4% PFA solution in a vented hood and incubated at room temperature (RT) for 10 min. The PFA was removed and cells were washed twice with 500 *μ*L of PBS, then permeabilized with 200 *μ*L of 0.2% (v/v) Tween 20 in PBS solution and incubated at RT for 5 min. Cells were washed twice with 200 *μ*L PBS at RT, leaving cells in PBS. They were then incubated with 100 *μ*L of* Texas Red-X Phalloidin* (2 U/mL, Life Technologies, USA) for 30 min at RT in agitation and washed twice with PBS. Nuclei were stained using DAPI (1 *μ*M, Life Technologies, USA) for 10 min at RT. Cells were mounted on a cover slide using ProLong Gold Antifade Reagent (Life Technologies, USA). All images were obtained using laser confocal microscope Leica TCS SP5 and a 63x oil objective 1.4NA (Leica Microsystems GmbH, Germany). Images were processed using LASAF 2.7.3v software (Leica Microsystems GmbH, Germany).

#### 2.5.5. Detection of NF-*κ*B p65 Subunit by Immunofluorescence

HT-29-NF-*κ*B-hrGFP E5 clone was seeded at 2 × 10^5^ cell/well in a 24-well plate containing glass cover slips in RPMI1640 supplemented with 10% (v/v) FBS. After 24 h, medium was renewed and TNF-*α* (50 ng/mL) was added and further incubated for 1 h. Culture media were removed, and immediately after, cells were fixed by adding 500 *μ*L of 4% PFA solution and incubated at RT for 10 min. The PFA was removed and cells were washed twice with 500 *μ*L of PBS. Cells were permeabilized with 200 *μ*L of 0.1% (v/v) Triton X-100 in PBS solution and incubated for 15 min at RT and then washed twice with 200 *μ*L PBS at RT, leaving cells in PBS. Cells were incubated with 200 *μ*L of 2% (v/v) BSA in PBS solution for 1 h at RT. Immediately, 200 *μ*L of primary antibody (Anti-NF-*κ*B-p65 ab7970, Abcam, USA) were added (final concentration 2 *μ*g/mL) and incubated ON at 4°C. The antibody was removed, and cells were washed once with 0.01% Tween 20 in PBS solution for 15 min and twice with PBS for 1 min. Afterward, 200 *μ*L of secondary antibody (Anti-rabbit Alexa 594 A11012, Life Technologies, USA) was added (1 : 1000 dilution) and incubated for 1 h in the dark at RT. Cells were washed once with 0.01% Tween 20 in PBS solution for 15 min and twice with PBS for 1 min. Nuclei were stained using Hoescht 33342 (1 *μ*M, Sigma). Cells were mounted on a cover slide using ProLong Gold Antifade Reagent. All images were obtained using laser confocal microscope Leica TCS SP5 equipped with a 63x oil objective 1.4NA. Images were processed using LASAF 2.7.3v software.

### 2.6. Validation of Reporter Cell Lines

#### 2.6.1. Lactobacillus and Reporter Cell Lines Coculture Assays

HT-29-NF-*κ*B-hrGFP clones were seeded in 48-well plates (1 × 10^5^ cells/well) in RPMI1640 supplemented with 10% (v/v) FBS. For Caco-2-NF-*κ*B-hrGFP C3 clone, 2 × 10^5^ cells were seeded in 24-well plates in DMEM containing 10% (v/v) FBS.* Lactobacillus reuteri* (ATCC 23272) and* Lactobacillus plantarum* (ATCC 8014) were grown ON at 37°C in MRS broth (Oxoid, UK) and then subcultured and harvested by centrifugation (5 min at 3,000 g). On coculture day, bacteria were washed twice with PBS buffer and resuspended in DMEM. A correlation curve between absorbance measured at 570 nm (*A*_570 nm_) versus colony forming units was constructed for each strain. The *A*_570 nm_ values were employed to calculate the bacterial number used in each experiment.

After 24 h cultivation of the reporter cell lines, bacteria were added and incubated for 2 h without the addition of antibiotics. Then, gentamicin (50 *μ*g/mL) and TNF-*α*, 5 ng/mL for Caco-2-NF-*κ*B-hrGFP C3 clone, or 1 ng/mL for HT-29-NF-*κ*B-hrGFP clones, were added. Cells were further incubated for 48 or 18–24 h for Caco-2-NF-*κ*B-hrGFP (C3) or HT-29-NF-*κ*B-hrGFP (E5 and F6) clones, respectively. Culture supernatants were collected, cleared by centrifugation at 3.000 g for 5 min, transferred into a new tube, frozen, and stored at −80°C for a maximum period of 1 month until IL-8 quantification. Cells were trypsinized and analyzed by flow cytometry as described previously. Cells without treatment and cells treated only with TNF-*α* or the lactobacilli were included as controls. Data was normalized against TNF-*α* controls (considered as 100%) and plotted as the mean ± SD of triplicates.

#### 2.6.2. IL-8 Quantification

The levels of the proinflammatory cytokine IL-8 were determined in the cell culture supernatants by flow cytometry using Flow Cytomix technology (eBioscience, USA). Briefly, this assay is based on a mixture of antibody-coated beads which specifically react with IL-8. Beads were incubated with the samples or the standard curve containing recombinant IL-8, and then a biotin-conjugated secondary antibody was added, which specifically binds the captured IL-8. Finally, Streptavidin-Phycoerythrin, which emits fluorescent signals, was added and 500 events were acquired by flow cytometry according to manufacturer recommendations. Flow Cytomix Pro Software version 3.0 was used for the analysis (eBioscience, USA).

#### 2.6.3. Anti-Inflammatory Natural Cyclic Peptide Culture Assays

Caco-2-NF-*κ*B-hrGFP C3 clone was seeded in 48-well plate (1 × 10^5^ cells/well) in DMEM containing 10% (v/v) FBS. HT-29-NF-*κ*B-hrGFP E5 and F6 clones were seeded in 96-well plates (2.5 × 10^4^ cells/well) in RPMI1640 supplemented with 10% (v/v) FBS. After 24 h, the natural peptide (10 ng/mL) and stimulus (5 ng/mL TNF-*α* for Caco-2-NF-*κ*B-hrGFP C3 clone, 1 ng/mL TNF-*α* for HT-29-NF-*κ*B-hrGFP E5 and F6 clones, and 5 ng/mL LPS only for HT-29-NF-*κ*B-hrGFP F6 clone) were added simultaneously. Cells were further incubated for 48 h for Caco-2 and 18–24 h for HT-29 reporter clones. Cells were trypsinized and analyzed by flow cytometry as described previously. Cells without treatment and cells treated only with the stimuli or the natural peptide were included as controls. Data was normalized against stimuli controls (considered as 100%) and plotted as the mean ± SD of triplicates.

### 2.7. Statistical Analysis

Data was expressed as the mean ± SD of triplicates and three independent experiments were executed. Statistic calculations were performed using the GraphPad Prism Software version 5.00 (Trial). Differences were considered statistically significant when *P* < 0.05 using One-Way ANOVA test with Dunnett's posttest.

## 3. Results and Discussion

Since its discovery in 1988, NF-*κ*B has been recognized as a key signaling system in response to immune and proinflammatory stimuli. The NF-*κ*B activation has direct applications in immunomodulatory strategies. Currently, direct screenings for drug discovery are being performed. Even though methods such as the detection of specific NF-*κ*B binding in a nuclear extract or visualization of nuclear p65 by microscopy are being used, these methods correlate with but do not directly prove transcriptional activation [13]. In this work, we report a functional assay for NF-*κ*B activation in IECs using the GFP reporter protein as read-out.

### 3.1. Generation of Stable Reporter Cell Lines

**Figure 1:**
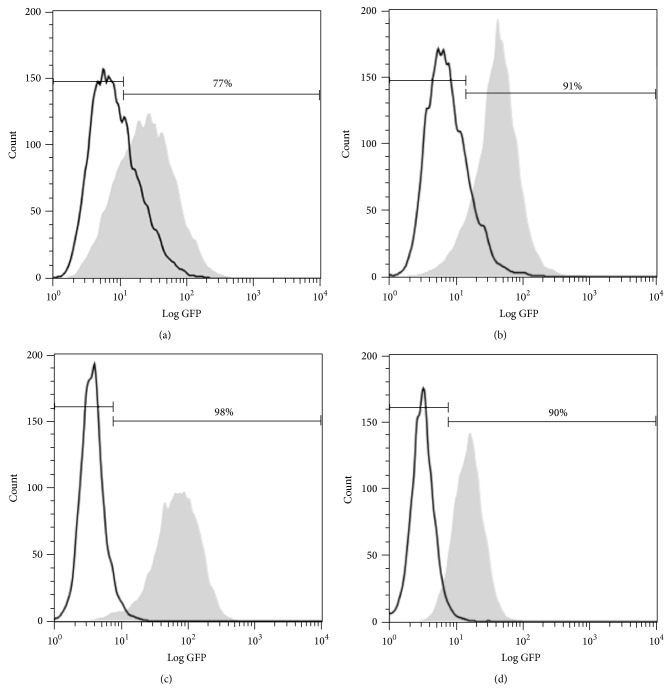
Response of Caco-2-NF-*κ*B-hrGFP D5 (a), C3 (b), HT-29-NF-*κ*B-hrGFP E5 (c), and F6 (d) clones to TNF-*α*. Clones were stimulated with 50 ng/mL TNF-*α* and GFP expression was evaluated by flow cytometry after 48 h. Cell population was gated using the FSC versus SSC dot plot and then represented in a GFP histogram plot. Untreated cells are shown in white while treated cells are shown in grey. The raw data of GFP positive population in treated cells is shown.

Caco-2 and HT-29 cell lines derive from human colon and were originally isolated from colorectal adenocarcinomas. Although they are of tumor origin, both of them are widely used as human IEC models [8, 14–16]. In order to have NF-*κ*B activation reporter IECs, Caco-2 and HT-29 cells were stably transfected with the pNF-*κ*B-hrGFP plasmid which contains the GFP gene under control of NF-*κ*B binding elements. Upon stimulation, NF-*κ*B will activate and translocate into the nucleus guiding the expression of the GFP. The use of GFP as reporter gene has some peculiarities: it does not need a substrate; it remains stable when subjected to heat, extreme pH, and chemical denaturants [17]. Moreover, it does not require cell lysis and its expression can be estimated simultaneously with cellular viability. After transfection, cells were selected in culture medium containing hygromycin B and the percentage of GFP positive (% GFP^+^) cells in response to TNF-*α* was evaluated in the resistant clones. The two best responding reporter clones derived from each cell line were selected for further characterization. The selection criterion was to choose those which displayed a high signal upon TNF-*α* stimulation and a high ratio between nonstimulated and stimulated states. The selected clones for Caco-2-NF-*κ*B-hrGFP were D5 and C3 and for HT-29-NF-*κ*B-hrGFP were E5 and F6. Representative histograms of each selected clone are shown in Figure 1.

### 3.2. Characterization of Reporter Cell Lines

In order to characterize whether the reporter gene in the selected clones reflects the regulation of the NF-*κ*B signaling pathway, we extensively characterized its time course kinetics response, its reaction to different concentrations of known NF-*κ*B modulating molecules, such as proinflammatory cytokines, and the stability of the reporter cell clones along the splitting passages.

#### 3.2.1. Time Course Kinetics

**Figure 2:**
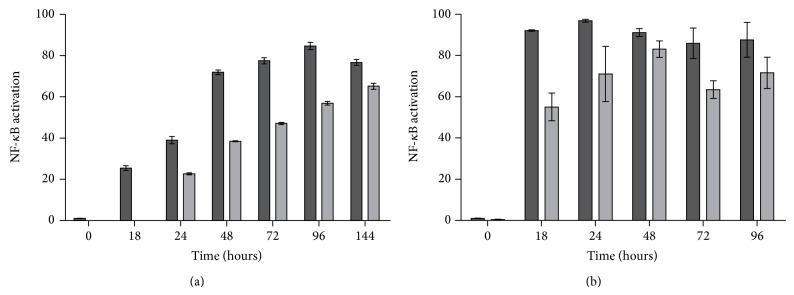
NF-*κ*B activation time course kinetics for different clones. Caco-2-NF-*κ*B-hrGFP C3 (black bar) and D5 (grey bar) clones (a) were seeded at 1 × 10^5^ cell/well, and HT-29-NF-*κ*B-hrGFP E5 (black bar) and F6 (grey bar) clones (b) were seeded at 5 × 10^4^ cell/well. Cells were cultured ON and treated with 50 ng/mL of TNF-*α* over time. Cells were trypsinized and GFP expression was analyzed at different time points by flow cytometry. Data was expressed as mean of triplicates with SD error bars. NF-*κ*B activation was determined by the subtraction of the % GFP^+^ of nonstimulated cells from the % GFP^+^ of stimulated cells.

We examined the kinetics of activation of the NF-*κ*B reporter systems by incubating the above-mentioned selected clones with TNF-*α* (50 ng/mL) at different times, from 18 to 144 h (Figure 2). Activity of the reporter Caco-2-NF-*κ*B-hrGFP C3 clone increased in a time dependent manner, with a maximum effect occurring at 96 h of continuous stimulation. On the other hand, clone D5 showed a slower NF-*κ*B activation, making it possible to detect GFP expression after 24 h of induction and continued to increase throughout the experiment (144 h). Both HT-29-NF-*κ*B-hrGFP clones showed a faster time course kinetics response, detecting GFP^+^ cells from 18 h. HT-29-NF-*κ*B-hrGFP E5 clone reached maximum expression at 18 h and remained stable until 96 h, while clone F6 exhibited its highest response at 48 h. Lakhdari and colleagues reported similar results when incubating the reporter clone HT-29/kb-seap-25 with TNF-*α*, which increased in a time dependent manner, with a maximum effect occurring after 24 h of stimulation [8]. In view of these results, 48 h was selected as the optimal time point for the execution of further experiments for Caco-2-NF-*κ*B-hrGFP C3 clone and between 18 and 24 h for HT-29-NF-*κ*B-hrGFP derived clones. Since Caco-2-NF-*κ*B-hrGFP D5 clone presented the lowest ratio between nonstimulated and stimulated states and in addition it showed slow time course kinetics, it was not further characterized.

#### 3.2.2. Activation of NF-*κ*B with Different Stimuli

**Figure 3:**
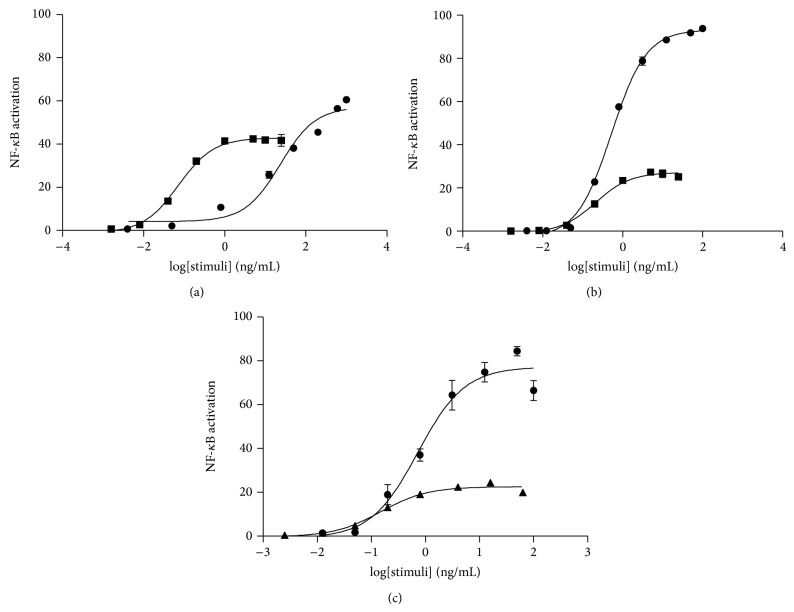
Activation of NF-*κ*B with different stimuli. Caco-2-NF-*κ*B-hrGFP C3 clone (a) was seeded at 1 × 10^5^ cell/well and 24 h later stimulated during 48 h with TNF-*α* (circle) or IL-1*β* (square). HT-29-NF-*κ*B-hrGFP E5 (b) or F6 clones (c) were seeded at 5 × 10^4^ cell/well and 24 h later were treated during 18–24 h with LPS (triangle), TNF-*α* (circle), or IL-1*β* (square). Cells were trypsinized and GFP expression was analyzed by flow cytometry. Data was expressed as mean of triplicates with SD error bars. Data was fitted using nonlinear regression 3-parameter fit using GraphPad Prism. NF-*κ*B activation was determined as previously described.

We extensively characterized the response of the selected clones to known NF-*κ*B modulating molecules (TNF-*α*, IL-1*β*, and LPS). TNF-*α* and IL-1*β* were able to induce expression of the reporter gene in Caco-2-NF-*κ*B-hrGFP C3 clone in a dose dependent manner (Figure 3(a)), while LPS did not cause GFP expression (data not shown). When incubated with TNF-*α*, GFP expression showed a linear range between 0.050 and 1,000 ng/mL (Table 1) but did not reach saturation even at the highest dose tested (1,000 ng/mL). Meanwhile, for IL-1*β* it showed linearity from 0.008 to 1.000 ng/mL (Table 1) and the response was saturated at 1.000 ng/mL (Figure 3(a)). Furthermore, TNF-*α* and IL-1*β* were able to activate GFP expression in HT-29-NF-*κ*B-hrGFP E5 clone (Figure 3(b)), showing linearity from 0.050 to 3.125 ng/mL and 0.008 to 0.200 ng/mL for TNF-*α* and IL-1*β*, respectively. Nonetheless, LPS was not able to activate the expression of the reporter protein in this clone. On the other hand, stimulation with TNF-*α* and LPS, but not IL-1*β*, in HT-29-NF-*κ*B-hrGFP F6 clone activated the expression of the reporter protein in a dose-response manner (Figure 3(c)). A linear response was observed when cells were stimulated with LPS, between 0.040 and 0.800 ng/mL. On the other hand, TNF-*α* produced a similar linear range than clone E5 (Table 1). When comparing the EC50 of the stimuli for the three analyzed clones we could say they were similar between them, except for TNF-*α* in Caco-2-NF-*κ*B-hrGFP C3 clone whose dose-response curve did not reach saturation; thus, it could not be determined (Table 1).

Our results for Caco-2 and HT-29 derived clones are in agreement with Lakhdari and colleagues, who reported that the induction with TNF-*α* than IL-1*β* was stronger for the reporter clone HT-29/kb-seap-25 and vice versa for Caco-2 [8].

As it was previously stated, HT-29-NF-*κ*B-hrGFP F6 clone, but not E5 clone, was able to respond to LPS. Lakhdari and colleagues demonstrated that HT-29 reporter cell line revealed a low expression of TLR4 [8], which is consistent with the low response obtained for clone F6 after treatment with LPS. The lack of response of E5 clone could be explained by random integration episode of the reporter gene into the cell line genome, which may have altered the expression of TLR4 or other proteins in its signaling cascade. These findings support the need to screen for different clones when a reporter cell line is under development. In the case of Caco-2-NF-*κ*B-hrGFP C3 clone, despite the fact that it has been reported that this cell line expresses TLR4, these cells did not respond after treatment with LPS, which is in agreement with the results reported by Lakhdari and colleagues [8]. Regarding the EC50, our reporter cell clones HT-29 were equally sensitive to TNF-*α* and IL-1*β* stimulation, as HT-29/kb-seap-25 [8]. Furthermore, Trask reported activation of NF-*κ*B-p65 in HeLa cells mediated by TNF-*α* with an EC50 value of 0.07 ng/mL, which is tenfold more sensitive than our HT-29 clones. On the other hand, the NF-*κ*B activation mediated by IL-1*β* in HeLa showed an EC50 of 0.31 ng/mL, which was similar to our findings (for both, Caco-2 and HT-29 derived clones) [6].

#### 3.2.3. Stability of the Reporter Cell Lines

**Figure 4:**
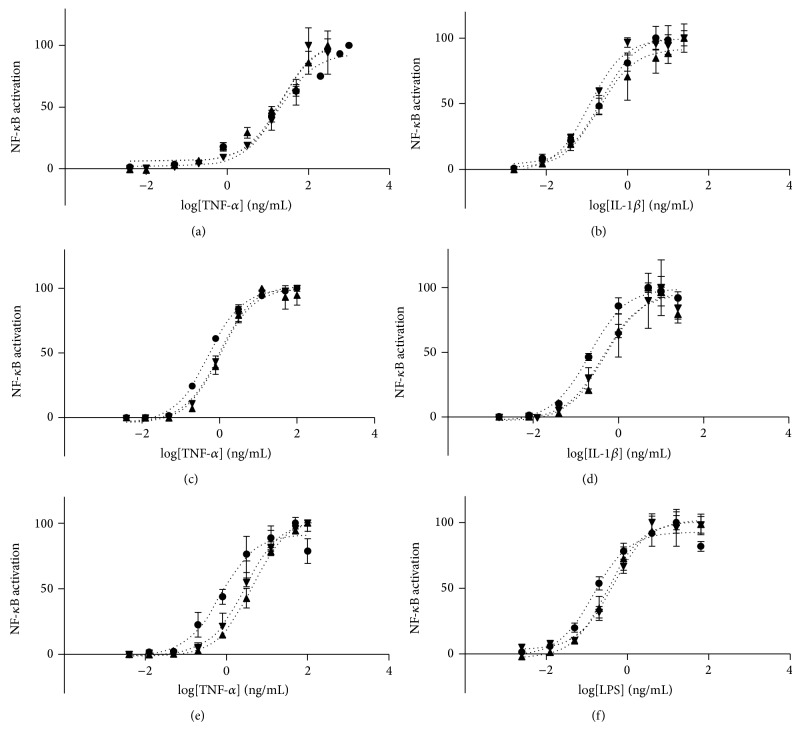
NF-*κ*B activation stability with passage number for different clones. Cells with 0 (black circle), 4 (downward triangle), or 8 (upward triangle) splitting passages of the reporter cell lines were seeded as follows: Caco-2-NF-*κ*B-hrGFP C3 clone ((a) and (b)) at 1 × 10^5^ cell/well and HT-29-NF-*κ*B-hrGFP E5 clone ((c) and (d)) or F6 clone ((e) and (f)) at 5 × 10^4^ cell/well. Cells were cultured ON and treated with TNF-*α*, LPS, or IL-1*β*. Cells were trypsinized and GFP expression was analyzed by flow cytometry. Data was normalized and fitted using nonlinear regression 3-parameter fit using GraphPad Prism. NF-*κ*B activation was determined as previously described.

It is critical to gauge the number of cell passages in the assay before a noticeable decline is observed. Cells with many passages may not survive, may become contaminated, or may fail to respond in the assay over time [6]. In order to verify reporter cell line stability in time, different passage numbers (passages 0, 4, and 8, which represent fresh thaw cells or cells that had undergone a weekly splitting for 1 or 2 months, resp.) were stimulated with TNF-*α*, IL-1*β*, and LPS. Dose-response curves were plotted for the clones with different stimuli (Figure 4) and EC50 were calculated and summarized in Table 2. For all the clones analyzed, the linear range of the dose-response curve did not change with the passage number. The EC50 remained unaltered for Caco-2-NF-*κ*B-hrGFP C3 clone when stimulated with IL-1*β*. Even though the *R* ^2^ values of the curve fitting were similar for both clones derived from HT-29-NF-*κ*B-hrGFP, there was a significant shift in the NF-*κ*B EC50 response to cytokines in cells with a high passage number. Based on these findings we recommend to use cells with as low a passage number as possible, and as a general rule, never exceed two months in culture (8 passages). Our results are in agreement with those obtained by Trask, who reported the NF-*κ*B response to TNF-*α* stimulation in HeLa cells with different cell splitting passage numbers [6]. He described a significant loss in responsiveness of NF-*κ*B translocation with an increase in calculated EC50 values, which directly correlated with increasing passage numbers.

#### 3.2.4. Polarization of Caco-2-NF-*κ*B-hrGFP Cells

Bacteria and compounds interact differently with polarized and nonpolarized epithelial cells. For example, flagellin activates IL-8 production in nonpolarized epithelial cells but only induces expression when added to the basolateral membrane [13]. It is therefore interesting to evaluate the Caco-2 reporter cell line using polarized cells, as it resembles the intestinal epithelium.

**Figure 5:**
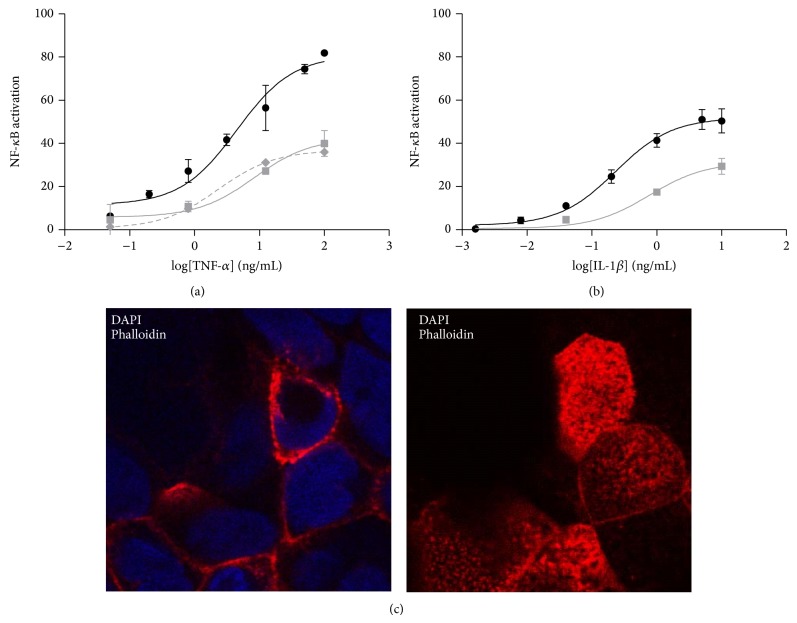
NF-*κ*B activation in polarized Caco-2-NF-*κ*B-hrGFP C3 clone cells. Polarized in plastic culture plates (grey continuous line), transwell filter (grey dotted line) and nonpolarized (black line) cells were stimulated during 48 h with TNF-*α* (a) or IL-1*β* (b). For polarization in transwell filter, only TNF-*α* was assayed and it was added in the basolateral chamber. Cells were trypsinized and GFP expression was analyzed by flow cytometry. NF-*κ*B activation was determined as previously described. Caco-2-NF-*κ*B-hrGFP C3 clone cells were polarized for 21 days and then fixed, permeabilized, and stained with* Texas Red-X Phalloidin* (for actin distribution, red) and DAPI (for nuclei localization, blue). All images were obtained through confocal microscopy. Representative cells of basal and apical membranes are shown in left and right images, respectively (c). Data was expressed as mean of triplicates with SD error bars.

Once cells were polarized in plastic culture plates, their response to different stimuli was evaluated. Cells were stimulated with TNF-*α* or IL-1*β* (Figures 5(a) and 5(b)) for 48 h and GFP expression was analyzed by flow cytometry. These cells responded in a dose dependent manner after being polarized, but to a lesser extent than nonpolarized cells. This could be explained by the fact that, in fully polarized Caco-2 cells, the TNF-*α* and IL-1*β* receptors are localized in the basolateral membrane and therefore not accessible to the stimuli added to the apical surface [18–21]. Thus, cells were also polarized in a permeable filter support for 21 days and stimulated with TNF-*α* (Figure 5(a)) in the basolateral chamber for 48 h. GFP expression was analyzed by flow cytometry. The same dose response curve was obtained for both polarized methods (plastic surface and permeable filter). Despite the fact that the response of both polarized cells is lower than that obtained with nonpolarized cells, the EC50 is not statistically different (data not shown).

To further confirm the cell polarization, the distribution of actin and localization of the nucleus after 21 days of polarization was assayed in Caco-2-NF-*κ*B-hrGFP C3 clone cells. Cells were stained with* Texas Red-X Phalloidin* to visualize actin distribution and DAPI for nuclei localization (Figure 5(c)). In polarized cells, actin mainly distributes on the periphery, creating a brush border as shown in the apical membrane, while the nucleus, as evidenced by the images, locates proximal to the basal membrane of the cells.

#### 3.2.5. Detection of NF-*κ*B p65 Subunit by Immunofluorescence

**Figure 6:**
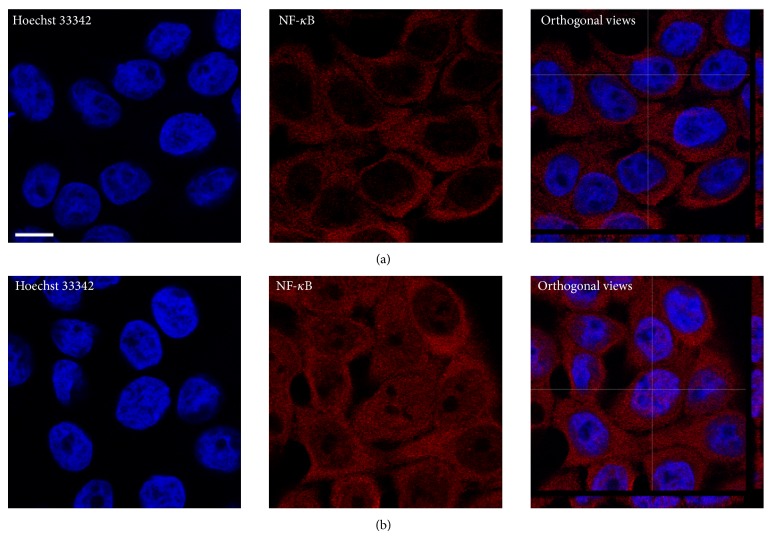
TNF-*α* induced NF-*κ*B translocation into the nucleus. Using an anti-p65 primary antibody, NF-*κ*B was detected in HT-29-NF-*κ*B-hrGFP E5 cells. Cells were stimulated with TNF-*α* (50 ng/mL) during 1 h, nonstimulated cells were used as control. Nuclei were stained with Hoescht 33342 (blue). Nonstimulated cells show only NF-*κ*B (red) in their cytoplasm (a); meanwhile, in stimulated cells NF-*κ*B is present both in the cytoplasm and nucleus (b). Scale bar = 10 *μ*m.

HT-29-NF-*κ*B-hrGFP E5 clone cells were stimulated with TNF-*α* for 1 h in order to correlate GFP expression with the translocation of NF-*κ*B into the nucleus. This translocation was visualized by confocal microscopy, labeling cells with an anti-p65 primary antibody (Figure 6). As evidenced by the images, in stimulated cells NF-*κ*B translocates to the nucleus, whereas in nonstimulated cells NF-*κ*B is detected only in the cytoplasm (secondary antibody control is shown in Supplementary Figure 1 in Supplementary Material available online at http://dx.doi.org/10.1155/2015/860534). This clone showed 95% of specific GFP^+^ cells (data not shown) after TNF-*α* induction, indicating a correlation between NF-*κ*B translocation and GFP expression in treated cells.

### 3.3. Validation of Reporter Cell Lines

NF-*κ*B reporter cells were constructed in view of performing screening of NF-*κ*B modulation capabilities within compound libraries or bacteria with potential therapeutic interest. The first validation assay was performed using the three selected reporter clones in coculture with LAB. The second step for validation was accomplished with a natural peptide obtained from a natural compound library.

#### 3.3.1. LAB and Reporter Cell Lines Coculture Assays

**Figure 7:**
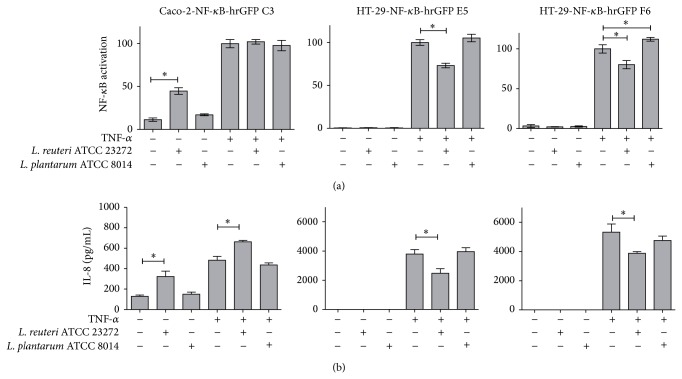
Caco-2-NF-*κ*B-hrGFP C3 and HT-29-NF-*κ*B-hrGFP E5 and F6 clones showed different NF-*κ*B activation (a) and IL-8 production (b) in response to LAB. Caco-2-NF-*κ*B-hrGFP C3 cells were seeded at 1 × 10^5^ cell/well, while HT-29-NF-*κ*B-hrGFP E5 and F6 clones were seeded at 5 × 10^4^ cell/well, cultured ON, and treated for 2 h with* L. reuteri* ATCC 23272 or* L. plantarum* ATCC 8014. Then, gentamycin and TNF-*α* (1 ng/mL) were added; 48 h or 18–24 h later (for Caco-2-NF-*κ*B-hrGFP C3 and HT-29-NF-*κ*B-hrGFP E5 and F6, resp.), cells were trypsinized and NF-*κ*B activation (measured by the % GFP^+^ cells) was analyzed by flow cytometry. Cell viability was over 90% for all tested conditions. Data was normalized against TNF-*α* control (considered as 100%). IL-8 quantification was performed in the harvested cell culture supernatant by flow cytometry. Results were expressed as the mean ± SD of triplicates of a representative experiment. ^*^ *P* < 0.05 using One-Way ANOVA with Dunnett's posttest.

Two LAB strains,* L. plantarum* ATCC 8014 and* L. reuteri* ATCC 23272, were selected based on immunomodulatory effects reported in literature on human monocyte-derived dendritic cells [10, 11, 22]. Since the health benefits of probiotics are highly dependent on the bacterial strain and each strain may contribute to host health through different mechanisms [23], it is important to test candidates in different cellular models. Caco-2-NF-*κ*B-hrGFP C3, HT-29-NF-*κ*B-hrGFP E5, and F6 clones were seeded, cultured ON, and treated for 2 h with* L. reuteri* ATCC 23272 or* L. plantarum* ATCC 8014. Then, gentamycin and TNF-*α* were added, and finally NF-*κ*B activation and IL-8 secretion were analyzed by flow cytometry. As depicted in Figure 7, Caco-2-NF-*κ*B-hrGFP C3 and HT-29-NF-*κ*B-hrGFP E5 and F6 clones showed different NF-*κ*B activation and IL-8 production in response to the added bacteria.* L. reuteri* ATCC 23272 decreased the TNF-*α*-induced NF-*κ*B activation in HT-29 clones with a concomitant reduction in IL-8 levels. These results are in accordance with Jones and colleagues who described a downmodulation of TNF-*α* production in LPS-activated monocytic THP-1 cells with other* L. reuteri* strains (ATCC PTA 6475 and ATCC PTA 5289) [23]. Nevertheless,* L. reuteri* ATCC 23272 was able to activate NF-*κ*B when it was cocultured with Caco-2-NF-*κ*B-hrGFP C3 clone and increased IL-8 levels either under nonstimulated or stimulated conditions. The different ability to detect NF-*κ*B modulation in the different reporter cell lines, for example with* L. reuteri* ATCC 23272, was already described by Lakhdari and colleagues using other stimuli [8]. On the other hand,* L. plantarum* ATCC 8014 did not show any immunomodulatory properties neither in Caco-2 nor HT-29 reporter cells (both in nonstimulated or stimulated conditions). Nonetheless, Cammarota and colleagues showed that another* L. plantarum* strain (DSMZ 12028) has probiotic attributes using IECs models [16]. These results emphasize the need for multiple models to assess the modulating properties of potential probiotic bacteria and reinforce the fact that the effectiveness of probiotics is strain-specific.

#### 3.3.2. Anti-Inflammatory Natural Cyclic Peptide

**Figure 8:**
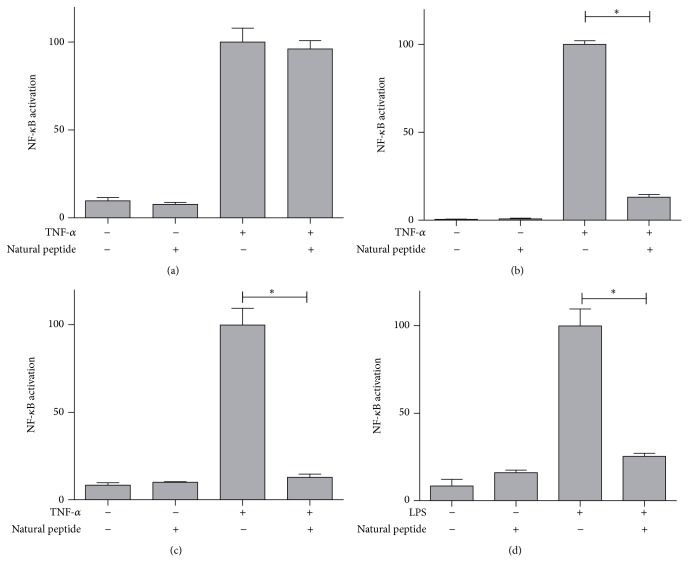
Natural cyclic peptide effect on NF-*κ*B activation induced by TNF-*α* and LPS on Caco-2-NF-*κ*B-hrGFP C3 and HT-29-NF-*κ*B-hrGFP E5 and F6 clones cells. The natural cyclic peptide was added to Caco-2-NF-*κ*B-hrGFP C3 clone (a) and HT-29-NF-*κ*B-hrGFP E5 (b) and F6 ((c), (d)) clones simultaneously with TNF-*α* ((a), (b), and (c)) or LPS (d), respectively. For HT-29-NF-*κ*B-hrGFP clones, 1 ng/mL TNF-*α* or 5 ng/mL LPS was employed, while for Caco-2-NF-*κ*B-hrGFP C3 clone 5 ng/mL TNF-*α* was used. After 48 h for Caco-2-NF-*κ*B-hrGFP clone and 18–24 h for HT-29-NF-*κ*B-hrGFP clones, NF-*κ*B activation (measured by the percentage of GFP^+^ cells) was analyzed by flow cytometry. Cell viability was over 90% for all tested conditions. Data was normalized against TNF-*α* or LPS controls (considered as 100%) and shown as the mean ± SD of triplicates of a representative experiment. ^*^ *P* < 0.05 using One-Way ANOVA with Dunnett's posttest.

In order to expand the uses of the reporter gene assay, we selected a natural cyclic peptide which was originally isolated from myxobacteria. This compound alters the function of the interferon pathway [12]. Caco-2-NF-*κ*B-hrGFP C3 clone was incubated for 48 h with the cyclic peptide in absence/presence of TNF-*α* (Figure 8(a)). HT-29-NF-*κ*B-hrGFP E5 and F6 clones were incubated for 18–24 h with the cyclic peptide in absence/presence of TNF-*α* for clone E5 (Figure 8(b)) and TNF-*α* or LPS for clone F6 (Figures 8(c) and 8(d)).

The peptide alone did not activate NF-*κ*B in any of the three reporter cell lines, while it was able to attenuate the NF-*κ*B activation induced by TNF-*α* and LPS in HT-29 reporter clones but not in the Caco-2 reporter clone. In the light of these results, further experiments will be conducted in order to determine the IL-8 concentration in culture supernatants.

In accordance with the coculture results, both HT-29 clones were able to sense the modulation of NF-*κ*B activation induced by the stimuli, showing the same trend. This suggests that the ability of the reporter cell lines derived from HT-29 to detect modulatory effect of NF-*κ*B activity is clone independent. Moreover, Caco-2 clone was unable to sense a modulatory effect of the natural peptide on NF-*κ*B activation. Based on these results, both reporter cell lines seem to have different capacity to detect modulation, which are in agreement with the findings reported by Lakhdari and collegues [8].

## 4. Conclusions

We have reported the generation, characterization, and validation of a cell-based screening system to study NF-*κ*B modulation in IECs and its first utilization within screening of LAB or a cyclic peptide derived from a natural compound library. Using the reporter gene strategy, we have obtained three reliable reporter cell clones (one from Caco-2 and two from HT-29) that allow a simple and rapid examination of NF-*κ*B activity regulation. The robustness of the selected clones was validated through their response to known activators, LAB and a natural peptide. We observed a dose dependent stimulation of NF-*κ*B in clones upon treatment with the proinflammatory cytokines TNF-*α* and IL-1*β* (Caco-2-NF-*κ*B-hrGFP C3 and HT-29-NF-*κ*B-hrGFP E5 clones) and TNF-*α* and LPS (HT-29-NF-*κ*B-hrGFP F6 clone). Furthermore, TNF-*α* and IL-1*β* were able to induce expression of the reporter gene also in polarized Caco-2-NF-*κ*B-hrGFP C3 clone, expanding the range of applications. We conclude that these new biological tools provide an alternative reporter system to the conventional existing one and have direct screening applications for synthetic and natural compound libraries or potential probiotics discovery.

## Supplementary Material

In absence of primary antibody anti-p65, no signal was detected in HT-29-NF-κB-hr-GFP E5 clone cells incubated with secondary antibody anti-rabbit Alexa 594. Immunofluorescence was performed as previously described in Materials & Methods.

## Figures and Tables

**Table 1:** Determination of EC50, detection limit, and linear range for different clones.

	Caco-2-NF-*κ*B-hrGFP		HT-29-NF-*κ*B-hrGFP			
	Clone C3		Clone E5		Clone F6	
	TNF-*α*	IL-1*β*	TNF-*α*	IL-1*β*	TNF-*α*	LPS
EC50 (ng/mL)	ND	0.23 (0.16–0.33)	0.52 (0.47–0.59)	0.21 (0.15–0.28)	0.72 (0.43–1.22)	0.15 (0.10–0.21)
Detection limit (ng/mL)	0.050	0.022	0.012	0.014	0.053	0.018
Linear range (ng/mL)	0.050–1,000	0.008–1.000	0.050–3.125	0.008–0.200	0.050–3.125	0.040–0.800

Different clones were seeded, cultured ON, and treated with TNF-*α*, LPS, or IL-1*β*. Cells were trypsinized and GFP expression was analyzed by flow cytometry. Data was normalized and EC50, detection limit, and linear range were calculated using nonlinear regression 3-parameter fit using GraphPad Prism. 95% confidence intervals are shown between brackets. ND: not determined.

**Table 2:** NF-*κ*B activation stability with passage number for different clones.

Passage number	Caco-2-NF-*κ*B-hrGFP		HT-29-NF-*κ*B-hrGFP			
	Clone C3		Clone E5		Clone F6	
	TNF-*α*	IL-1*β*	TNF-*α*	IL-1*β*	TNF-*α*	LPS
0	ND	0.23 (0.16–0.33)	0.52 (0.47–0.59)	0.21 (0.15–0.28)	0.72 (0.43–1.22)	0.15 (0.10–0.21)
4	ND	0.12 (0.09–0.16)	1.03 (0.77–1.38)	0.47 (0.30–0.75)	2.80 (2.58–3.08)	0.45 (0.32–0.63)
8	ND	0.20 (0.12–0.33)	1.00 (0.86–1.16)	0.44 (0.23–0.81)	4.30 (3.72–0.81)	0.33 (0.24–0.44)

Different cell splitting passages of the reporter cell lines were seeded. Cells were cultured ON and treated with TNF-*α*, LPS, or IL-1*β*. Cells were trypsinized and GFP expression was analyzed by flow cytometry. Data was normalized and EC50 (ng/mL) was estimated using nonlinear regression 3-parameter fit using GraphPad Prism. 95% confidence intervals are shown between brackets. ND: not determined.
