# Supplementary figures and images for: HT-29 and Caco-2 Reporter Cell Lines for Functional Studies of Nuclear Factor Kappa B Activation
Source: Mediators Inflamm. 2015 Mar 10;2015:860534. doi: 10.1155/2015/860534 (PMC4377483; doi:10.1155/2015/860534)

***Supplementary Figure 1***

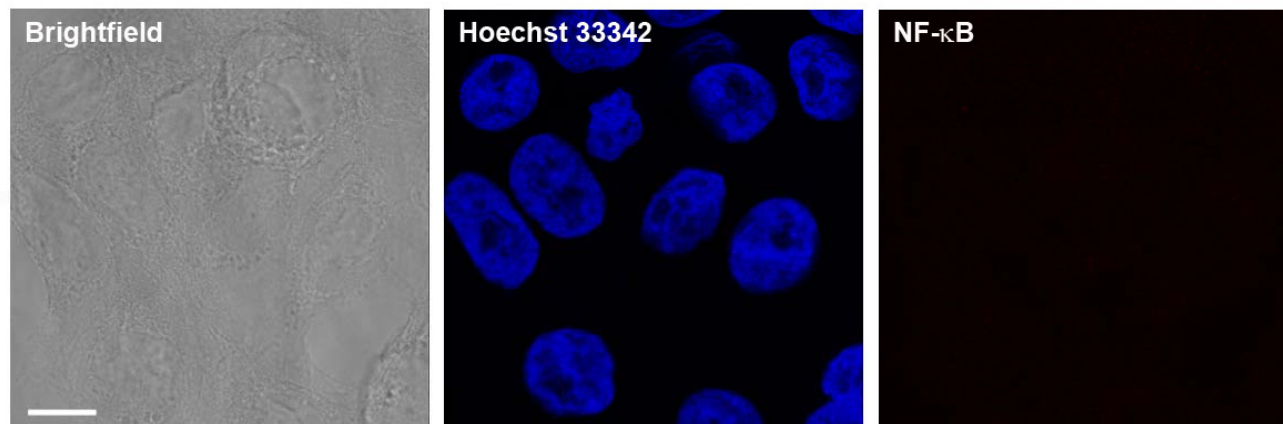

Supplement: Supplementary file 1 — In absence of primary antibody anti-p65, no signal was detected in HT-29-NF-κB-hr-GFP E5 clone cells incubated with secondary antibody anti-rabbit Alexa 594. Immunofluorescence was performed as previously described in Materials & Methods. [file 860534.f1.pdf]
